# A chromosome‐level genome of mango exclusively from long‐read sequence data

**DOI:** 10.1002/tpg2.20441

**Published:** 2024-03-10

**Authors:** Upendra Kumari Wijesundara, Ardashir Kharabian Masouleh, Agnelo Furtado, Natalie L. Dillon, Robert J. Henry

**Affiliations:** ^1^ Queensland Alliance for Agriculture and Food Innovation University of Queensland Brisbane Queensland Australia; ^2^ Department of Agriculture and Fisheries Mareeba Queensland Australia; ^3^ ARC Centre of Excellence for Plant Success in Nature and Agriculture University of Queensland Brisbane Queensland Australia

## Abstract

Improvements in long‐read sequencing techniques have greatly accelerated plant genome sequencing. Current de novo assemblies are routinely achieved by assembling long‐read sequence data into contigs that are assembled to chromosome level by chromatin conformation capture. We report here a chromosome‐level mango genome using only PacBio high‐fidelity (HiFi) long reads. HiFi reads at high coverage (204x) resulted in the assembly of 17 chromosomes, each as a single contig with telomeres at both ends. The remaining three chromosomes were represented each by two contigs, with telomeres at one end and ribosomal repeats at the other end. Analyzing contig ends allowed them to be paired and linked to generate the remaining three complete chromosomes, telomere‐to‐telomere but with ribosomal repeats of uncertain length. The assembled genome was 365 Mb with 100% completeness as assessed by Benchmarking Universal Single‐Copy Orthologs analysis. The haplotypes assembled demonstrated extensive structural differences. This approach using very high genome coverage may be useful for assembling high‐quality genomes for many other plants.

## Core Ideas

- High genome coverage and high‐fidelity (HiFi) sequencing method alone facilitated high‐quality mango genome assembly.
- The assembled mango genome reported the highest possible completeness (100%) and contiguity (contig N50: 15 Mb).
- Phased haplotypes showed extensive structural variations.

**Abbreviations**
BUSCO: Benchmarking Universal Single‐Copy Orthologs
CLC‐GWB: CLC Genomic Workbench
CLR: continuous long‐read
CTAB: cetyltrimethylammonium bromide
Hi‐C: chromatin conformation capture
HiFi: high‐fidelity; KEGG, Kyoto Encyclopedia of Genes and Genomes
ONT: Oxford Nanopore Technologies
rRNA: ribosomal RNA
T2T: telomere‐to‐telomere

## 1 INTRODUCTION

High‐quality reference genomes and annotations are fundamental to characterize structural and functional variations in genomes and to explore the mechanisms of important traits facilitating modern molecular breeding. As a result of the development and continued improvement of single‐molecule long‐read sequencing technologies, we can now assemble highly accurate telomere‐to‐telomere (T2T) genomes. The era of de novo genome assembly began with Sanger sequencing, whereas the first assembled eukaryotic genome was *Saccharomyces cerevisiae* in 1996 (Dujon, 1996). Subsequently, genomes of many other species were assembled, including rice (Goff et al., 2002), maize (Schnable et al., 2009), *Arabidopsis* (The Arabidopsis Genome Initiative, 2000), and human (Venter et al., 2001). Subsequent advances in next‐generation sequencing have further improved the plant genome assemblies, while they still exhibited thousands of gaps within the pseudomolecules, primarily due to the high prevalence of repetitive sequences and limitations in read lengths (75–300 bp) (Belser et al., 2021; Chen et al., 2023).

The continuous long‐read (CLR) sequencing and nanopore sequencing developed by Pacific Biosciences (PacBio) and Oxford Nanopore Technologies (ONT), respectively, revolutionized genome analysis. They may generate long reads (>10 kb), spanning numerous repetitive elements within genomes. However, these technologies had comparatively higher error rates (5%–20%). Enhancements in read accuracy of nanopore sequencing (up to 95%–99%) (Wang et al., 2021) and the introduction of PacBio high‐fidelity (HiFi) sequencing capable of generating 99.9% accurate long reads (15–25 kb) (Cheng et al., 2021) established these technologies as the gold standard for generating high‐quality genomes. These new long‐read sequencing technologies have resulted in enhanced quality and contiguity of assemblies for both model organisms and plants with complex genomes (Choi et al., 2020; Pham et al., 2020). However, the long‐read data are often complemented with other techniques such as optical mapping, chromatin conformation capture (Hi‐C), genetic maps, or hybrid approaches to scaffold and orient the contigs to reach chromosome‐scale T2T genomes (Kong et al., 2023). With the recent release of T2T assembly of human X chromosome (Miga et al., 2020), the first T2T human genome was released, which used a combination of nanopore sequencing, HiFi sequencing, linked read sequencing from 10x Genomics, and optical mapping (Nurk et al., 2022). In recent years, T2T‐assembled genomes have been reported for various plant species, including rice (Huang, 2023), maize (Chen et al., 2023), banana (Belser et al., 2021; Liu et al., 2023), kiwifruit (Yue et al., 2023), and watermelon (Deng et al., 2022), by using scaffolding methods to assembly contigs from long‐read sequences to chromosome level. A recent banana genome used nanopore long reads plus Illumina short reads (Belser et al., 2021), while the other banana genome assembly has incorporated HiFi and nanopore reads along with Hi‐C data (Liu et al., 2023). T2T genome assemblies for rice (Huang, 2023) and watermelon (Huang, 2023) have used HiFi, nanopore reads, and Hi‐C data, whereas the maize genome (Chen et al., 2023) has used Illumina reads together with both long‐read sequencing approaches.

Mango (*Mangifera indica*) is a diploid species (2*n* = 40) and one of the most economically successful fruits cultivated in tropical and subtropical regions of the world. Despite having great economic significance, the knowledge of mango genetic resources is limited. Several mango genomes have been assembled in recent years. First, the Indian cultivar Amrapali was assembled with Illumina reads. The large assembly size (492 Mbp) achieved compared to the estimated genome size (439 Mbp) indicated the redundancy in the assembly (Singh et al., 2021). In 2020, cultivar Alphonso was assembled using PacBio long reads, Illumina, and Hi‐C reads leading to an improved 392 Mb genome (Wang et al., 2020). Three other mango cultivars were also assembled concurrently (Bally et al., 2021; Li et al., 2020; Ma et al., 2021), while the Amrapali genome was being improved by PacBio long‐read sequencing (Singh et al., 2021). The Alphonso was considered as the reference genome for mango since compared to other genomes, it provided high contiguity and completeness. In this study, we report a breakthrough achievement: the development of a high‐quality mango genome solely using HiFi data. This assembly represents the first genome for mango where all the chromosomes have telomeres at both ends, signifying a remarkable advancement in the field of genome assemblies.

**Core Ideas**

- High genome coverage and high‐fidelity (HiFi) sequencing method alone facilitated high‐quality mango genome assembly.
- The assembled mango genome reported the highest possible completeness (100%) and contiguity (contig N50: 15 Mb).
- Phased haplotypes showed extensive structural variations.

## 2 MATERIALS AND METHODS

### 2.1 Sample collection, DNA extraction, and sequencing

Young fresh leaves of *M. indica* ‘Irwin’ was sourced from a tree located at the Walkamin Research Station, Mareeba (17°08 02″S and 145°25 37″E), North Queensland, Australia. DNA extraction was carried out according to a cetyltrimethylammonium bromide (CTAB) method (Kilby & Furner, 2002) with modifications. Modifications optimized the protocol, resulting in high‐quality DNA. Note that 0.5 g of finely pulverized leaf tissue was mixed with 6 mL of CTAB I buffer (2% CTAB, 1.4 M NaCl, 100 mM Tris HCl, 20 mM EDTA, and pH 8.0) pre‐heated at 67°C. The sample was incubated in a water bath at 67°C for 15–20 min with occasional inversions. Note that 10 µL of RNase A (10 mg/mL) was added and incubated for 10 min at room temperature; 0.5 volume of chloroform was added and centrifuged at 2000 *g* for 5 min. The upper phase was transferred to a new tube, and one‐tenth of CTAB II (10% CTAB, 0.7M NaCl) buffer was added and mixed. Sample was extracted again with 0.5 volume of chloroform. Without reducing the salt concentration in the aqueous phase, DNA was precipitated by adding 0.6 volume of isopropanol and centrifuging at 4800 *g* for 10 min. The supernatant was discarded, and 5 mL of 70% ethanol was added to the pellet and centrifuged at 4800 *g* for 10 min. After removing the supernatant, the pellet was dried, and the DNA was re‐dissolved in 10 mM Tris HCl buffer (pH 8.0). After assessing the quality and quantity of the extracted DNA, PacBio HiFi sequencing was carried out in two PacBio Sequel II SMRT cells at the Institute for Molecular Bioscience, The University of Queensland, Australia.

### 2.2 RNA extraction and sequencing

Young leaf and pre‐ and post‐anthesis flower tissues were sourced from the tree located at the Walkamin Research Station, Mareeba (17°08 02″S and 145°25 37″E), North Queensland, Australia. RNA was extracted using a CTAB method (Wang & Stegemann, 2010) with modifications and purified with the Qiagen RNeasy Mini Kit (Qiagen). RNA extracted from different tissues was sequenced separately using Illumina short‐read sequencing at the Australian Genome Research Facility, University of Queensland.

### 2.3 Draft contig level genome assembly

The quality of the sequenced PacBio HiFi reads was assessed using SMRT Link v11.0. HiFi reads were assembled by HiFiasm Denovo assembler, a fast haplotype resolved de novo assembler (Cheng et al., 2021) with default settings to assemble heterozygous genomes with built‐in duplication purging parameters. The high‐performance computer facility at the University of Queensland was used to run the assembly. The contiguity of the assembled collapsed genome and two haplotypes (hap1 and hap2) were assessed using the quality assessment tool v5.2.0 (Gurevich et al., 2013). The completeness of the assemblies was assessed using Benchmarking Universal Single‐Copy Orthologs (BUSCO) using the viridiplantae database (BUSCO v 5.4.6) (Simão et al., 2015), which is the closest database to the genome assembly. However, genomes published previously have been analyzed using Eukaryota and Embryophyta databases. Therefore, to compare the completeness of our genome, we also performed the BUSCO analysis using the Embryophyta and Eukaryota databases. Raw Illumina reads (paired‐end) were imported to the CLC Genomic Workbench (CLC‐GWB) and quality trimmed at 0.01 quality limits. K‐mer analysis was carried out in Jellyfish (v2.2.10) (Manekar & Sathe, 2018), and the results were further analyzed with GenomeScope v2.0 (http://genomescope.org/genomescope2.0) (Ranallo‐Benavidez et al., 2020).

### 2.4 Assembly of pseudomolecules/chromosomes and telomere sequence identification

Pseudochromosomes were identified and characterized in terms of telomeric repeats and ribosomal RNA (rRNA) repeats. Reference‐based contig anchoring method was used to anchor contigs onto chromosomes (Li et al., 2021). D‐GENIES (Cabanettes & Klopp, 2018) was used to align the contig‐level assembly with the published *M. indica* ‘Alphonso’ chromosome‐scale genome (Cabanettes & Klopp, 2018), and the contigs were sorted and oriented with respect to *M. indica* genome. The contigs corresponding to 20 chromosomes were identified and assigned to 20 chromosomes.

TIDK v0.2.1 (https://github.com/tolkit/telomeric‐identifier) was used to find telomeres in the contigs using the normalized and unified sequence “AAACCCT/TTTGGGA,” and the telomere sequence was also checked manually. For the contigs that had telomeres at one end only, nucleotide sequence of the other end was confirmed with the NCBI nucleotide blast. Based on the NCBI blast results, three contigs that were already assigned to chromosomes (chromosomes 8, 11, and 19) had rRNA repeats. Also, three smaller contigs, which had telomeric repeats only at one end, also had similar types of ribosomal repeats. The presence of the repetitive sequences in the six contigs were also confirmed by checking them manually, and they were assigned to chromosomes 8, 11, and 19. In the conditions where two contigs should be linked in order to get a complete chromosome, they were joined manually by 100 N's in between to imply the two contigs joined and to represent the possibility of missing some more repeats in between. Furthermore, contigs relevant to 20 chromosomes of the haplotypes were also selected by aligning the assemblies of haplotypes against the Irwin collapsed genome in D‐GENIES (Cabanettes & Klopp, 2018). Telomeric repeats of the contigs belonging to two haplotypes were identified by TIDK v0.2.1, and contigs were joined to obtain complete chromosomes. More information on the method of joining contigs is provided in the Supporting Information.

In collapsed assembly, 20 chromosomes and other smaller contigs (0.16–1 Mb), which were not assigned to chromosomes, were aligned with *M. indica* ‘Irwin’ chloroplast genome assembled by Get Organelle pipeline v.1.7.5 using Illumina reads (Jin et al., 2020). Thereby, the presence of chloroplast sequences in small set of contigs was analyzed. Smaller contigs (0.16–1 Mb) were also aligned with *M. indica* mitochondrial genome (CM021857.1) and rRNA sequences (5S, 5.8S, 18S, and 28S) (downloaded from NCBI) to analyze the presence of ribosomal repeats in smaller contigs.

### 2.5 Genome comparison and synteny analysis

The MUMer software (Marçais et al., 2018) was used for alignment comparison analysis with parameters (maxmatch ‐c 100 ‐b 500 ‐l 50). Genome comparison was conducted between Irwin collapsed genome and Alphonso genome (CATAS_Mindica_2.1), Irwin collapsed genome and hap1 and hap2 separately, and finally between Irwin hap1 and hap2. The alignments were filtered using the delta‐filter implemented in Mummer with the parameters (‐m ‐i 90 ‐l 100). The structural variations and sequence differences analysis was performed using the Synteny and Rearrangement Identifier (Goel et al., 2019) and the results were visualized using plots (Goel & Schneeberger, 2022).

### 2.6 Genome annotation

After developing the chromosome‐level assemblies for the collapsed genome as well as for two haplotypes, all three genomes were passed through genome annotation. Repeat elements were identified with de novo approach using Repeatmodeler2 v2.0.4 (Flynn et al., 2020) and masked with Repeatmasker v.4.1.5 (Chen, 2004) using the softmasking option. Quality and adapter‐trimmed RNA sequencing reads of leaf and flower tissues were aligned to the soft‐masked genomes using HISAT2 tool (Kim et al., 2019). Structural features were inferred using Braker3 v.3.0.3 (Brůna et al., 2021; Gabriel et al., 2023). Annotation completeness was assessed with BUSCO analysis.

Functional annotation was performed in Omicsbox 3.0.30 (OmicsBox, 2019). The CDS sequences were passed through BLASTX homology search with *viridiplantae* taxonomy against the NCBI non‐redundant protein database with the maximum number of 20 hits and *e*‐value of 1.0E‐3. Gene ontology (GO) terms were assigned for the hits obtained by BLAST search via mapping and Blast2go annotation. CDS sequences were also passed through InterProScan and eggNOG mapper. All the GO terms retrieved via InterProScan were merged into the already existing Blast2go annotations. GOs from EggNog mapper annotations were also merged with the GO retrieved from InterProScan and Blast2go annotations. GO annotations were validated based on the True‐Path‐Rule by removing all redundant terms for a given sequence. CDS sequences that had no blast hits were extracted and ran through a coding potential assessment. *Arabidopsis thaliana* model, which was already built, and *M. indica* model created using coding and non‐coding sequences were used for the coding potential assessment. Kyoto Encyclopedia of Genes and Genomes (KEGG) pathway analysis (Kanehisa & Goto, 2000) was conducted in Omicsbox v.3.0.30 to identify important biosynthesis pathways in mango.

### 2.7 Identification of unique and shared genes among collapsed genome and two haplotypes

The Irwin collapsed genome and two haplotypes were used to find the unique genes in each genome. Protein sequences of genomes were clustered at *e*‐value of 1e‐2 using OrthoFinder algorithm in OrthoVenn3 (Sun et al., 2023). Unique clusters were identified for each genome, and accordingly, unique genes for each genome were extracted. KEGG pathway analysis (Kanehisa & Goto, 2000) was conducted in Omicsbox v.3.0.30 to identify genes related to important biosynthesis pathways, and important biological processes and cellular processes related to these genes were identified from functional annotation results.

In collapsed assembly, 20 chromosomes and other smaller contigs (0.16–1 Mb), which were not assigned to chromosomes, were aligned with the assembled *M. indica* chloroplast genome with Get Organelle pipeline v.1.7.5 (Jin et al., 2020). Thereby, the incorporation of chloroplast sequences into nuclear genome and presence of chloroplast sequences in small set of contigs were analyzed. Smaller contigs (0.16–1 Mb) were also aligned with *M. indica* mitochondrial genome (CM021857.1) and rRNA sequences (5S, 5.8S, 18S, and 28S) (downloaded from NCBI) to analyze the presence of ribosomal repeats in smaller contigs.

## 3 RESULTS

### 3.1 Genome sequencing and assembly

The total HiFi yield was 74.74 Gb (204x coverage) (Table S1). The genome assembly performed with HiFiasm (Cheng et al., 2021) generated a collapsed assembly and two phased haplotypes. The collapsed assembly and haplotypes haplotype 1 (hap1) and haplotype 2 (hap2) were composed of a total of 4642, 4711, and 1515 contigs, respectively. BUSCO analysis detected 100% of the single copy orthologs with a contig N50 of 14.98 Mb for collapsed assembly. Furthermore, hap1 and hap2 covered 98.1% and 99.0% single‐copy orthologs with contig N50 of 13.21 Mb and 15.45 Mb, respectively (Table S2). K‐mer analysis estimated 1.24% heterozygosity and 365 Mb genome size (Figure S1).

### 3.2 Highly contiguous assembly of Irwin genome

**FIGURE 1:**
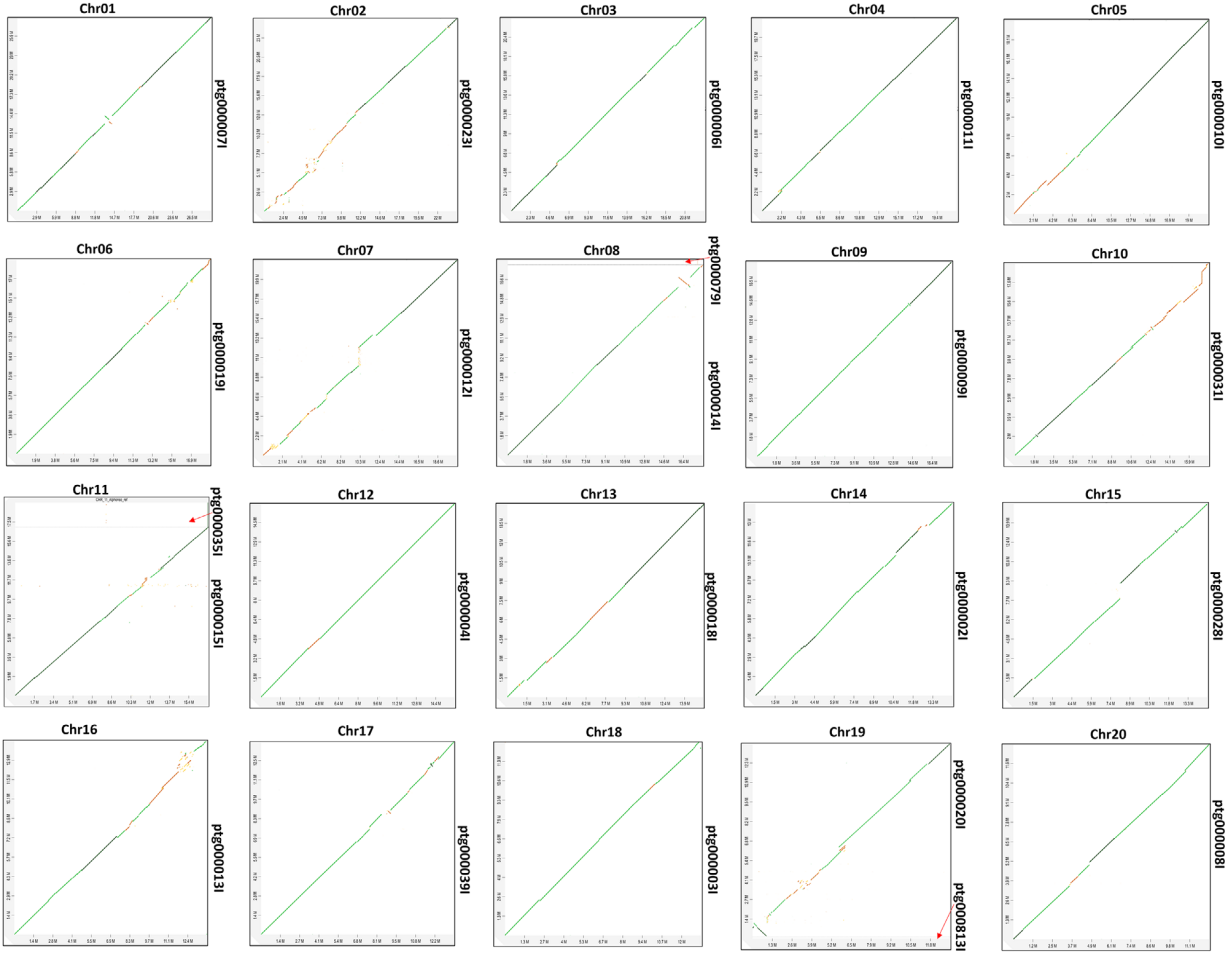
Individual chromosome‐scale pseudomolecules of Irwin collapsed genome aligned with the respective chromosomes of the published *Mangifera indica* (cv. Alphonso) genome. The *x*‐axis indicates the chromosomes of the Alphonso genome, and the *y*‐axis indicates the corresponding contigs in our assembly. Chromosome names/numbers were given with respect to the Alphonso genome. Out of 20, 17 chromosomes were represented by single contigs, while the remaining three chromosomes consisted of two contigs. Since the Alphonso genome lacked telomeres, possibly including a part of genomic region in chromosomes 8, 11, and 19, the smaller contigs with telomeric repeats in our assembly did not align with the respective chromosomes of the Alphonso genome, as shown in the dot plot.

After aligning Irwin collapsed assembly using Minimap2 v2.24 (Li, 2018), all the contigs were sorted and re‐oriented with respect to the Alphonso genome so that chromosome numbers could be assigned (Figure S2). According to the dot plot, single contigs were aligned with each of 19 chromosomes, except for chromosome 11 which had two contigs (Figure 1). The two contigs of chromosome 11 had telomeres at one end and 18S rRNA repeats at the other end (Table S3). The presence of the same type of repetitive sequence allowed the two contigs to be linked by 100 Ns.

Although only one contig was identified for chromosomes 8 and 19 (ptg000014l and ptg000020l, respectively) in the dot plot, both contigs had telomeres only at one end. However, the Alphonso is not a T2T genome and lacks 26 telomeres, including the telomeres of the respective ends in chromosomes 8 and 19 (Table S4). Therefore, contigs corresponding to chromosomes 8 and 19 with telomeres at one end did not align with the Alphonso genome because the respective ends in the Alphonso chromosomes 8 and 19 are incomplete. However, TIDK v0.2.1 identified three smaller contigs having telomeres (ptg000079l, ptg000813l, and ptg002605l).

The contigs ptg000014l and ptg000079l had 18S rRNA repeats at one end and telomeres at the other end, confirming that ptg000079l belonged to chromosome 8. The two contigs were linked by 100 Ns to get a complete pseudomolecule corresponding to chromosome 8. Similarly, ptg000020l and ptg000813l also had rRNA repeats at one end, which spanned 18S rRNA, ITS1, 5.8S rRNA, ITS2, and 26S rRNA gene sequences, and telomeres at the other end. These two contigs were also joined to develop a pseudomolecule for chromosome 19 (Table S3). Selection of the contigs for chromosomes 8 and 19 was also based on long‐read alignment of respective contigs in CLC‐GWB (Figure S3), which was then confirmed manually by checking for the presence of repetitive sequences at the ends of contigs joined together. In this way, we developed the collapsed genome (365 Mb) (Table 1) for Irwin consisting of 23 contigs (Figure 1; Table S5), which had telomeres at both ends of all 20 chromosomes. The remaining 4619 contigs ranged between 0.16 and 1.0 Mb, and most of these contigs showed high similarity to the chloroplast, mitochondrial genome sequences, and to the nuclear rRNA genes (Figure S4).

**TABLE 1:** Chromosome and telomeric repeat lengths in the collapsed genome and two haplotypes.

	Collapsed			Hap1			Hap2		
Chr. number	Chr. length (bp)	Length of telomere repeat at start (bp)	Length of telomere repeat at the end (bp)	Chr. length (bp)	Length of telomere repeat at start (bp)	Length of telomere repeat at the end (bp)	Chr. length (bp)	Length of telomere repeat at start (bp)	Length of telomere repeat at the end (bp)
1	28,791,319	9465	10,818	28,791,319	9465	10,818	27,822,494	4869	12,866
2	25,591,877	9077	1847	22,006,409	5536	0	25,591,877	9077	1847
3	22,617,554	12,411	9018	22,617,554	12,411	9018	21,915,563	7744	9075
4	21,893,517	4082	8640	21,770,901	12,066	10,325	21,111,872	4115	9451
5	20,095,946	14,402	6736	20,095,946	14,402	6736	20,115,022	7704	6948
6	18,861,676	11,585	8151	18,861,676	11,585	8151	18,367,742	3704	5679
7	22,062,971	9661	6539	21,480,999	9353	6465	22,190,909	7164	6507
8	18,467,756	7688	16,886	18,467,756	7688	16,886	18,165,562	7964	16,401
9	18,285,638	9488	13,469	18,285,638	9488	13,469	17,257,157	9412	2838
10	19,503,959	17,050	7486	17,061,531	6872	8100	19,503,959	17,050	7486
11	19,477,525	13,601	7941	19,471,163	13,404	8050	16,582,699	8178	0
12	16,091,751	8003	18,832	16,091,751	8003	18,832	15,803,983	12,286	15,996
13	14,984,773	8513	5708	14,984,773	8513	5708	14,854,956	8667	7398
14	14,459,570	6313	12,164	14,459,570	6313	12,164	14,055,478	6363	7573
15	15,449,518	10,211	4031	12,370,655	10,327	4460	15,449,518	10,211	10,211
16	14,365,229	7916	14,578	14,365,229	7916	14,578	13,355,921	7154	9917
17	13,854,346	13,077	4233	13,854,346	13,077	4233	12,156,532	8137	10,210
18	13,214,860	7334	7715	13,214,860	7334	7715	12,798,669	11,103	10,197
19	13,611,789	9596	15,429	13,051,374	9596	15,589	15,311,864	17,162	15,310
20	12,942,385	5257	11,374	12,942,385	5257	11,374	12,209,353	5232	8535
Total	364,623,959	194,730	191,595	354,245,835	188,606	192,671	354,621,130	173,296	174,445

Abbreviation: Hap, haplotype.

**FIGURE 2:**
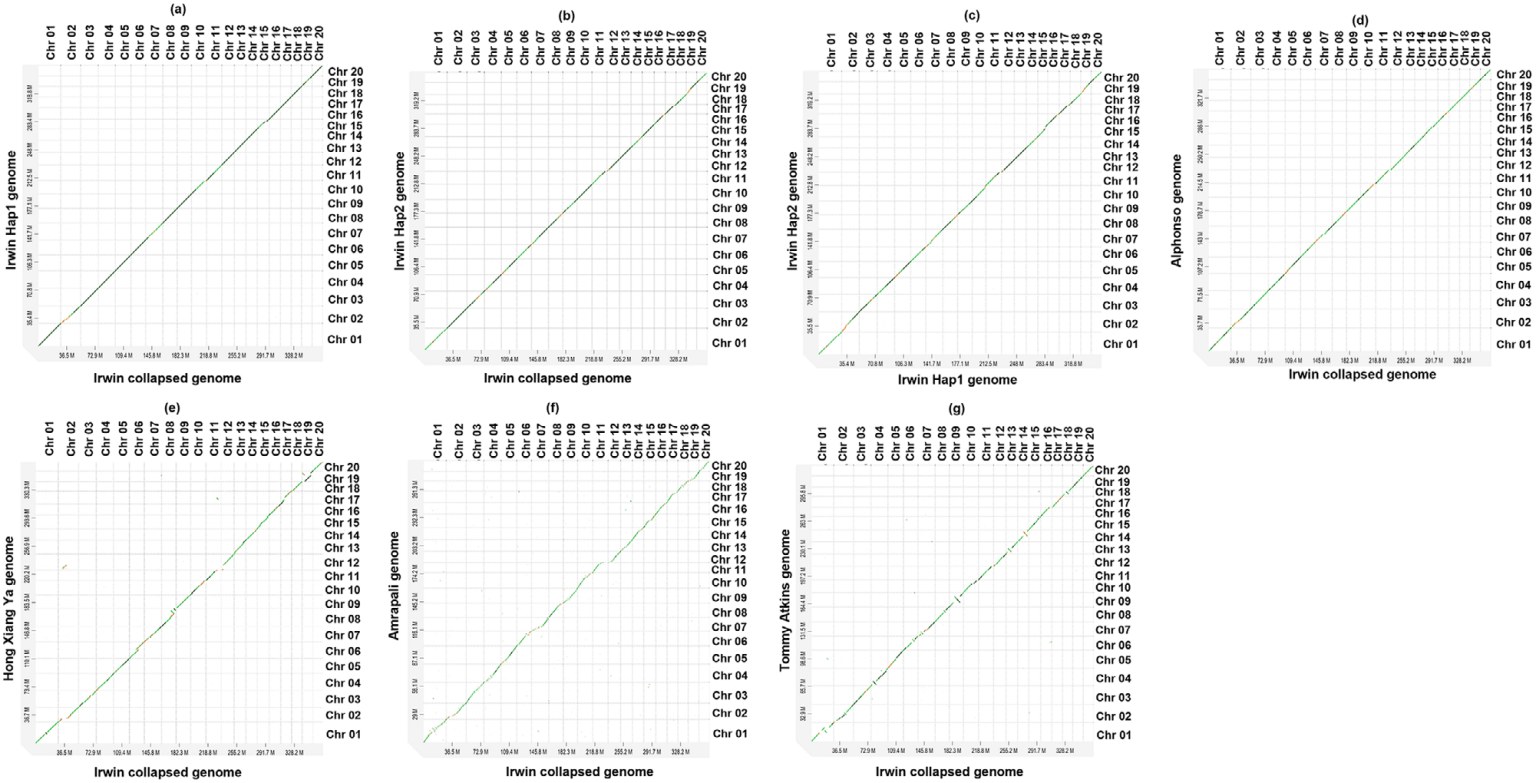
Dot plots show the alignment between the Irwin collapsed, hap1, and hap2 genomes and the synteny between the 20 chromosomes of the current Irwin genome and previously published *Mangifera indica* genomes. (a): Irwin collapsed genome versus hap1 genome, (b): Irwin collapsed genome versus Irwin hap2 genome, (c): Irwin hap1 versus hap2 genomes, (d): Irwin collapsed and Alphonso genome, (e): Irwin collapsed and Hong Xiang Ya genome, (f): Irwin collapsed and Amrapali genome, and (g): Irwin collapsed and Tommy Atkins genome.

Two phased haplotype assemblies were aligned with the Irwin collapsed genome (Figure S1), and contigs belong to same chromosome were linked (Supporting Information). The hap1 and hap2 assemblies were less contiguous, requiring 39 and 34 contigs, respectively. However, 14 and 11 chromosomes were covered each by a single contig in hap1 and hap2. In both haplotypes, 19 chromosomes had telomeres at both ends, while only one chromosome had telomeres at one end. The hap2 had a larger genome size compared to hap1, but the collapsed genome exceeded both haplotypes (Figure 2a–c; Table 1).

Currently, all five published mango genomes have been sequenced by either Illumina or PacBio CLR sequencing with (Ma et al., 2021; Wang et al., 2020) or without (Bally et al., 2021; Li et al., 2020; Singh et al., 2021) integrating Hi‐C data. The genome we assembled only with HiFi reads is highly contiguous compared to previously published genomes (Figure 2d–f), increasing contig N50 up to 14.98 Mb. The published Irwin genome did not provide any information about telomeres (Ma et al., 2021). TIDK v0.2.1 revealed that none of the chromosomes in Tommy Atkins genome have telomeres, while the other genomes have only 10–14 telomeres (Table 2). Here, we developed a highly contiguous mango genome for Irwin. Out of 20, 17 chromosomes were assembled T2T, and the remaining three chromosomes (chromosomes 8, 11, and 19) also had telomeres at both ends, although each chromosome consisted of two contigs, indicating three gaps in the assembled genome.

**TABLE 2:** Comparison of *Mangifera indica* genome assemblies.

Genomic feature	This study (Irwin)	Amrapali (Singh et al., 2021)	Hong Xiang Ya (Li et al., 2020)	Alphonso (Wang et al., 2020)	Tommy Atkins (Bally et al., 2021)	Irwin (Ma et al., 2021)
Genome size (with unplaced scaffolds) (Mb)	–	411	371	392	375	396
Genome size (20 chromosomes only) (Mb)	365	290	367	357	328	375
Coverage (x)	204	290	388	240	180	62
Contig N50 (Mb)	14.98	0.98	4.82	3.50	0.0411	1.03
Number of telomeres	40	10	14	10	0	–
**Complete BUSCO (%)**						
Eukaryota (*N* = 2326)	99.1	93.6	–	–	–	94
Embryophyta (*N* = 1614)	99.2	–	93.3	95.9	97.4	–
Viridiplantae (*N* = 425)	100	90.6	97.6	96.0	96.0	
Sequencing platform	PacBio HiFi	PacBio; Illumina, BioNano	PacBio CLR; Illumina	PacBio CLR; Illumina, Hi‐C	Illumina Hiseq	PacBio CLR; Illumina, Hi‐C
Assembly tool	HiFiasm	FALCON	FALCON FALCON‐unzip	Canu	DeNovo Magic	MECAT

Abbreviations: BUSCO, Benchmarking Universal Single‐Copy Orthologs; CLR, continuous long‐read; Hi‐C, chromatin conformation capture; HiFi, high‐fidelity; PacBio, Pacific Biosciences.

### 3.3 Genome comparison and synteny

**FIGURE 3:**
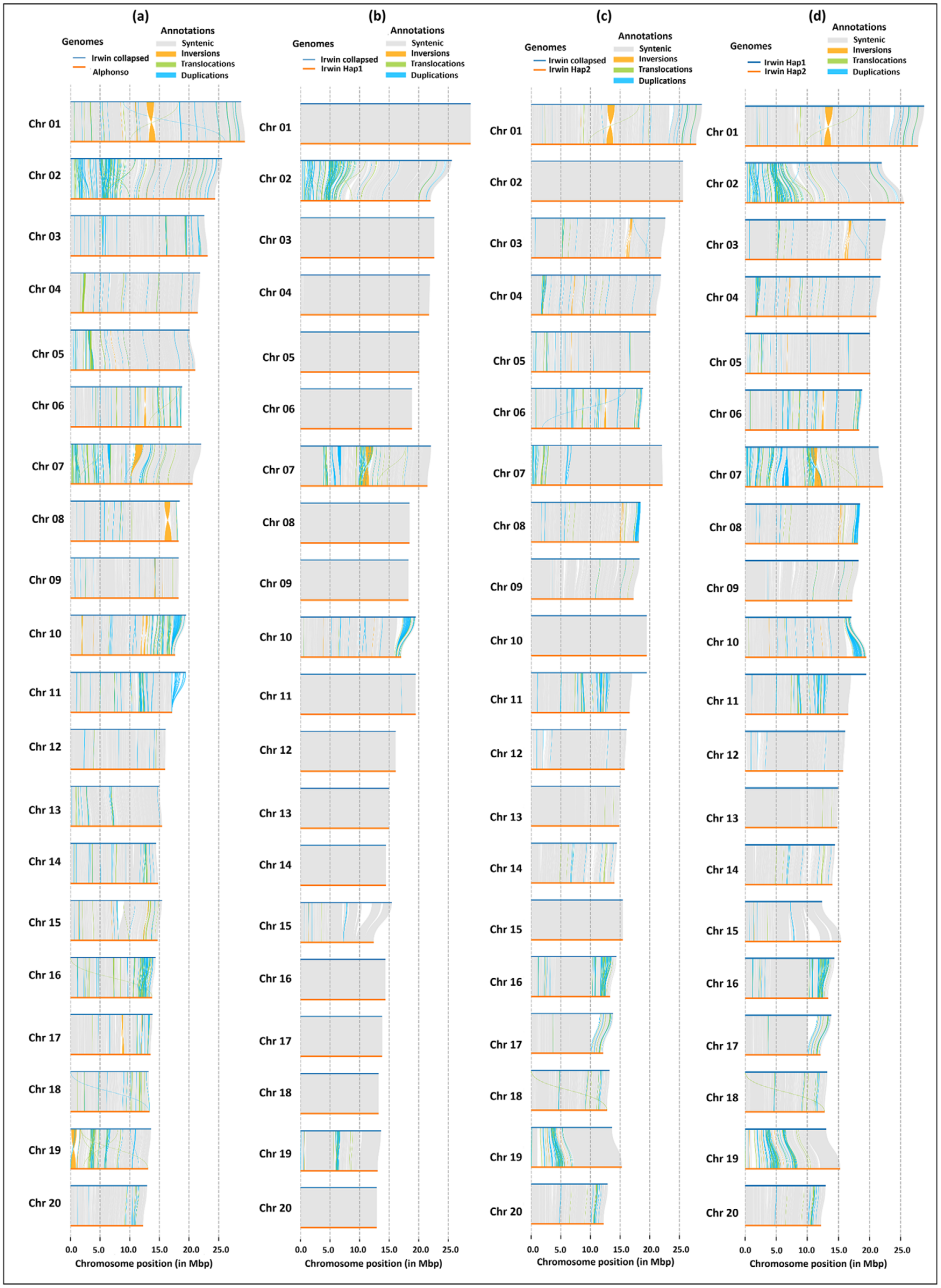
Chromosome‐wise structural variations between *Mangifera indica* cultivars (a) Irwin collapsed genome versus Alphonso genome (b) Irwin collapsed genome versus Irwin hap1, (b) Irwin collapsed genome versus Irwin hap2, and (c) Irwin hap1 versus Irwin hap2. Genome synteny between the *M. indica* ‘Irwin’ collapsed genome and the Alphonso genome identified a total of 69 inversions (6.4 Mb) and 2589 translocations (15.9 Mb). Also, a total of 8966 (29.9 Mb) and 5895 (17.1 Mb) duplications were identified in Irwin and Alphonso genomes, respectively. Comparison of the collapsed Irwin genome with the haplotype genomes identified 17 inversions and 412 translocations between the collapsed genome and hap1, whereas between the collapsed genome and hap2, 48 inversions and 1448 translocations were identified. A higher number of inversions and translocations were observed between the Irwin hap1 and hap2 genomes (65 and 2193, respectively).

Structural variations between Irwin and Alphonso genome, Irwin collapsed, and hap1 and hap2 genomes separately and between Irwin hap1 and hap2 genomes were identified (Figure 3). In the Irwin collapsed genome, sequence lengths of 13 chromosomes were identical to those of hap1, while the other three were identical to hap2. The comparison between hap1 and hap2 for these 16 chromosomes showed that the selected chromosome for collapsed genome from hap1 or hap2 has a higher sequence length. Of the remaining four, chromosomes 4, 11, and 19 were similar to hap1. However, chromosome 7 was a combination of hap1 and hap2, which had a higher chromosome length than that of both haplotypes (Figure 3; Table S6).

### 3.4 Repetitive elements analysis, gene prediction, and annotation

**FIGURE 4:**
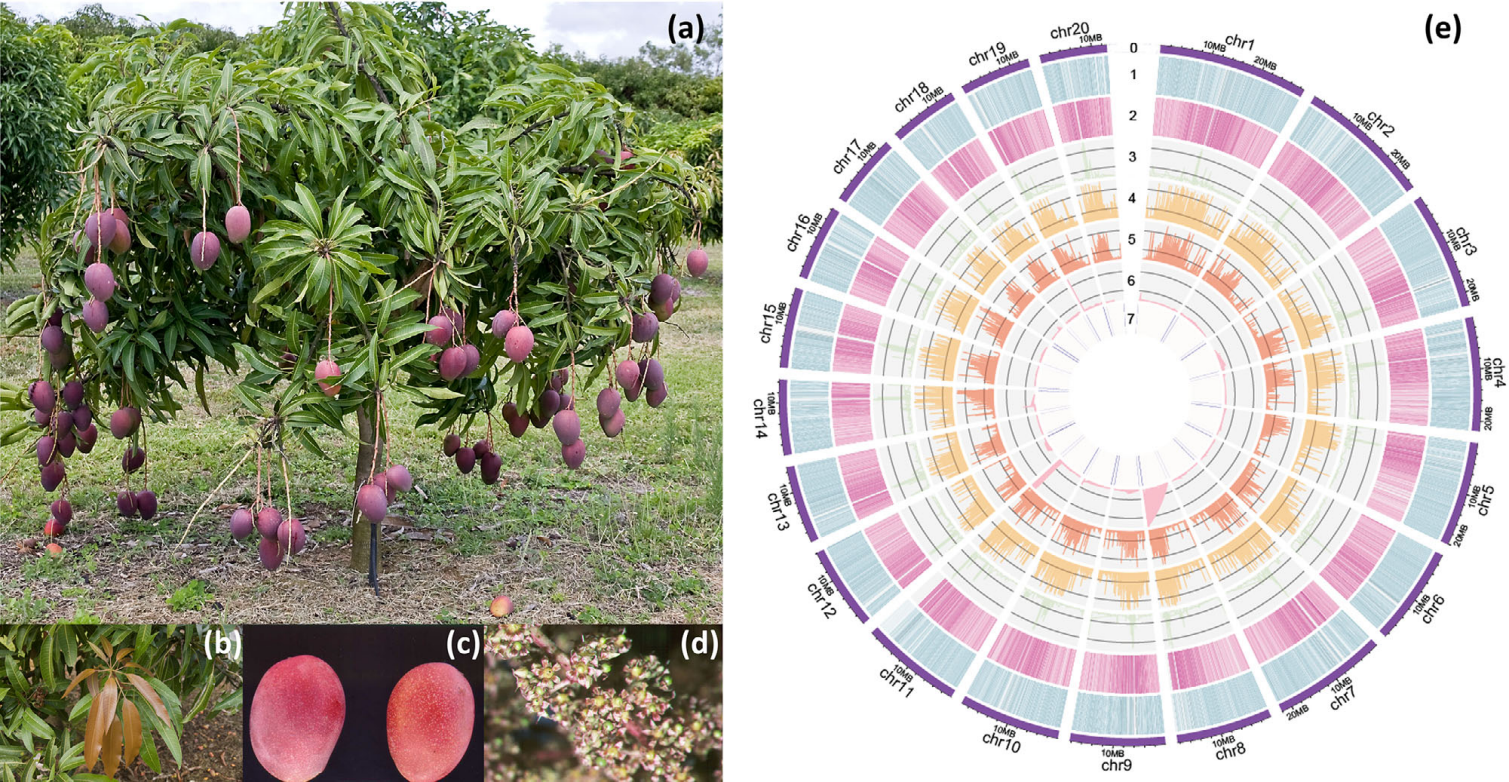
*Mangifera indica* ‘Irwin’ plant and circos plot to represent repetitive regions, telomeres, and genes in the genome. (a) Irwin tree, (b) young leaves, (c) fruit, (d) flower buds, (e) circos plot: (0) 20 pseudochromosomes (Mb), (1) predicted genes (2) Regions of DNA TE elements; (3) Regions of LINEs; (4) LTR Copia elements; (5) Regions of LTR Gypsy elements; (6) Regions of ribosomal RNA (rRNA), tRNA, and snRNA repetitive regions; and (7) Telomeric repeats. In the collapsed genome, a large proportion (46.33%, 168.9 Mb) was composed of interspersed repeats, and 29.63% of the repeats were unclassified. DNA transposons accounted for 4.24% (1.54 Mb) from which MULE‐MuDR (1.91%) and hobo‐Activator (1.08%) were the two major types. Among the classified retroelements, LINEs covered 0.86% of the genome, but LTR elements were the major type of repeats (11.51%), where 6.50% and 4.72% were covered by LTR Tp1/Copia and LTR Gypsy/DIRS1 elements, respectively. Small RNA (0.59%), simple repeats (1.12%), and low‐complexity repeats (0.22%) represented a minor proportion of the repetitive sequences.

We focused solely on the 20 chromosomes in all three genomes for annotations, since BUSCO results were identical for both the entire assembly and the 20 chromosomes. In total, we identified 177.61 Mb (48.71%), 171.90 Mb (48.53%), and 171.97 Mb (48.50%) repetitive sequences in Irwin collapsed, hap1, and hap2 genomes, respectively. Among classified repeats, LTR repeats were the most abundant repetitive elements (Table S7; Figure 4). RNA‐seq reads from leaf (30.5 Gb) and flowers (30.1 Gb) were equivalent to 154x coverage. A total of 35,220, 34,659, and 33,230 genes were identified in the collapsed, hap1, and hap2 genomes, with 42,973, 42,268, and 40,947 protein‐coding sequences (Table S2). The highest and the lowest number of genes were reported in chromosomes 1 and 20, respectively, in all three genomes (Table S8). In this study, the collapsed Irwin genome annotation showed a higher number of protein‐coding genes than published Tommy Atkins (26,616) (Bally et al., 2021), Hong Xiang Ya (34,529) (Li et al., 2020), and Alphonso (32,071) (GCF_011075055.1) genomes, but a smaller number than the published Irwin (36,756) genome (Ma et al., 2021). The collapsed, hap1, and hap2 genomes had 76.12%, 76.03%, and 77.33% functionally annotated genes (Figure S5), and most protein‐coding sequences with no BLAST hits have coding potential (Figure S6).

## 4 DISCUSSION

Combining highly accurate long‐read DNA sequencing technologies and effective assembly tools is critical for developing high‐quality genomes. However, the quality of extracted DNA for efficient long‐read sequencing is also important, and the presence of impurities diminishes the sequencing efficiency, affecting genome coverage, quality, and assembly completeness (Sharma et al., 2022). Mango leaves hold high levels of polysaccharides, polyphenols, proteins, and other secondary metabolites. Therefore, we used a CTAB method (Kilby & Furner, 2002) with modifications that enabled the isolation of high‐quality DNA, facilitating efficient long‐read sequencing.

Development of long‐read sequencing methods greatly improved the contiguity of the genome assemblies, and HiFi reads are the first type of data achieving the advantages of both higher read length (>10 kb) and accuracy (99.9%). In this approach, the subreads from a single polymerase read are linked computationally to generate a HiFi consensus read. This enables the generation of high‐quality reads preventing read overlaps, which may introduce errors due to incorrect overlaps (especially for repetitive regions) (Li et al., 2021). Comparative analysis of PacBio CLR and HiFi‐based *Macadamia* and Avocado assemblies have revealed that HiFi reads result in less fragmented assemblies compared to CLR reads (Sharma et al., 2021). Our Irwin genome assembly also confirmed that HiFi reads generate highly contiguous genomes as compared to currently assembled genomes with CLR reads (Amrapali and Hong Xiang Ya) and also compared to genomes (Irwin and Alphonso), which have used CLR reads and Hi‐C data. Generally, for plant genomes with 700–1000 Mb, HiFi read coverage of 40x has been considered sufficient to develop high‐quality genomes (Sharma et al., 2021), while increased sequence data further improves the assembly contiguity, generating more accurate assemblies (Sharma et al., 2021; Sharma et al., 2022). The Irwin collapsed genome we assembled with HiFi reads exhibits the highest completeness and contig N50 ever achieved for a mango genome, confirming that higher coverage and HiFi data facilitate high‐quality genomes.

To date, one of the challenges in developing high‐quality plant genomes has been the presence of high numbers of repetitive elements covering 30%–85% of the genomes (Schnable et al., 2009; Zhou et al., 2023). Among these, the presence of hundreds of tandemly arranged 5S rRNA and 45S rRNA (containing 18S, 5.8S, and 28S rRNA) genes makes it especially difficult to assemble a continuous sequence. The presence of these repeats near the end of the chromosomes prevents assembling telomeres (Sharma et al., 2022). The T2T banana genome assembled five out of 11 chromosomes each by single contig. The remaining six chromosomes required two to eight contigs, where most of the gaps between the contigs were located in the regions of 5S and 45S rRNA clusters (Belser et al., 2021). Similarly, Irwin collapsed genome we assembled consisted of 17 chromosomes, each represented by a single contig. The remaining three chromosomes required two contigs that had 45S rRNA clusters at the ends that required joining, confirming the difficulty of assembling rRNA repeats. Although the two haplotypes were less contiguous than the collapsed genome, the contigs in chromosomes 7, 8, 11, and 19 also had 45S rRNA clusters at the ends of the contigs that required joining.

Currently, the T2T genomes assembled have used HiFi reads and nanopore reads with or without Hi‐C data (Chen et al., 2023; Deng et al., 2022; Liu et al., 2023). Although the T2T banana genome used only nanopore sequencing (Belser et al., 2021), Illumina reads and optical mapping were used for assembly polishing and validation. The genome we assembled here for *M. indica* ‘Irwin’ may be the first plant genome assembled with HiFi reads alone, which had telomeres at both ends of all chromosomes. This is also the first mango genome assembled with HiFi reads showing the highest completeness (BUSCO = 100%) and contiguity (contigN50 = 14.9 Mb) achieved so far for a mango genome with only three gaps in chromosomes 8, 11, and 19 corresponding to ribosomal gene repeats. Compared to the Alphonso, this genome allowed the annotation of 3149 more genes. However, the previously published Irwin genome has a higher number of protein‐coding genes, possibly due to annotating pseudogenes and partial genes. Although the Irwin collapsed genome had all the telomeres, there was an additional contig in the contig assembly, which had telomeric repeats at one end. Since the two haplotypes are quite different, especially in terms of lengths, not all telomeres might be included in the collapsed genome when collapsing such haplotypes to one sequence.

Kiwifruit (Yue et al., 2023) and banana (Liu et al., 2023) genomes have been published as haplotype‐resolved T2T genomes, and they have used HiFi, ONT reads, and Hi‐C data for the assembly and phasing. However, we were able to generate a highly contiguous mango genome with all 40 telomeres only with HiFi sequence data, where each haplotype also had almost all the telomeres. The assembly and comparative analysis of the two phased haplotypes of Irwin allowed the investigation of structural variations, including insertions, deletions, duplications, and translocations that exist between the two haplotypes, providing a valuable resource for genome evolutionary studies as well as for allele‐specific expressions studies. Analysis of unique and shared gene families among collapsed, hap1, and hap2 genomes identified the presence of unique genes (Figure S7) involved in important biosynthesis pathways, biological processes, and molecular functions (Figure S8). Unique genes were also identified in the collapsed genome, probably due to the identification of more genes during annotation, which might not have been captured in annotating the haplotypes. Collapsing two very different haplotypes may reveal genes that have been missed at the haplotype level or introduce artifacts.

Finally, we conclude that highly contiguous pseudo‐haploid genome development is now feasible with HiFi sequence data alone. Here, the assembly of high‐coverage HiFi reads generated from high‐quality DNA is crucial in obtaining genomes with almost all the chromosomes represented by a single contig with all the telomeres. The high‐quality mango genome generated provides a valuable resource for advanced research focusing on important traits to improve mango breeding programs.

## AUTHOR CONTRIBUTIONS

**Upendra Kumari Wijesundara**: Conceptualization; data curation; formal analysis; investigation; methodology; writing—original draft; writing—review and editing. **Ardashir Kharabian Masouleh**: Conceptualization; data curation; formal analysis; methodology; software; supervision; writing—original draft; writing—review and editing. **Agnelo Furtado**: Conceptualization; data curation; methodology; resources; supervision; writing—original draft; writing—review and editing. **Natalie L. Dillon**: Conceptualization; investigation; project administration; resources; supervision; writing—review and editing. **Robert J. Henry**: Conceptualization; funding acquisition; methodology; project administration; resources; supervision; writing—review and editing.

## CONFLICT OF INTEREST STATEMENT

The authors declare no conflicts of interest.

## Supporting information


**Supplemental Figure S1** K‐mer profile (K = 17) spectrum generated in the Genome Scope for M. indica cv. ‘Irwin’.
**Supplemental Figure S2** Alignment of (a) M. indica cv. Alphonso genome vs ‘Irwin’ collapsed assembly, (b) ‘Irwin’ collapsed genome (20 chromosomes) vs ‘Irwin’ hap1 assembly, (c) ‘Irwin’ collapsed genome (20 chromosomes) vs ‘Irwin’ hap2 assembly.
**Supplemental Figure S3** Alignments between contigs in chromosomes 8, 11, and 19 conducted in CLC‐GWB.
**Supplemental Figure S4** Alignment of smaller contigs ranged between 16–1000Kb to chloroplast genome (a), mitochondrial genome (b), and rRNA repeats (c).
**Supplemental Figure S5** Summary of functional annotation for (a) collapsed, (b) Hap1, (c) Hap2 genomes and top hit species distribution for BLAST hits in (d) collapsed, (e) Hap1, (f) Hap2 genomes.
**Supplemental Figure S6** The coding potential assessment of CDS sequences that did not give a blast hit during functional annotation in the collapsed genome, hap1, and hap2 using (a) *Arabidopsis thaliana*, (b) *Manifera indica* and as model species.
**Supplemental Figure S7** Venn diagram displaying the shared and unique gene clusters among ‘Irwin’ collapsed genome, Hap1, and Hap2.
**Supplemental Figure S8** Functions of unique genes obtained from gene family analysis with related to biological process and molecular function.
**Supplemental Table S1** Summary of sequence data generated by two PacBio SMRT cells.
**Supplemental Table S2** Assembly and annotation statistics of *M. indica* cv. ‘Irwin’.
**Supplemental Table S3** Details of the repetitive sequences present in contigs corresponding to Chromosome 8, 11, and 19.
**Supplemental Table S4** Summary of telomeric repeats present in the reference genome currently available for mango (*M. indica* cv. Alphonso).
**Supplemental Table S5**: Details of contigs in the collapsed genome and two haplotypes.
**Supplemental Table S6**: Details of chromosomes selected from haplotypes to obtain collapsed assembly.
**Supplemental Table S7**: Repetitive elements in the collapsed genome and two haplotypes.
**Supplemental Table S8** Number of genes in chromosomes of collapsed genome and haplotypes.
**Supplemental Methods**: Joining contigs in Irwin haplotype 1 and haplotype 2.

## Data Availability

All the raw sequencing reads (PacBio HiFi, Illumina, and RNA sequencing) have been deposited in the NCBI Sequence Read Archive database (BioProject: PRJNA1034099, BioSamples: SAMN38055185, SAMN38051351, and accession IDs: SRR26666709, SRR26668549, SRR26643579, SRR26637609, SRR26637683). The whole genome assembly and annotation have been deposited in the Genome Warehouse in the National Genomics Data Centre, Beijing Institute of Genomics, Chinese Academy of Sciences (accession ID GWHEQCT00000000, BioProject PRJCA020898, Biosample SAMC3141886).
