# Supplementary material for: A chromosome‐level genome of mango exclusively from long‐read sequence data
Source: Plant Genome. 2024 Mar 10;17(2):e20441. doi: 10.1002/tpg2.20441 (PMC12806855; doi:10.1002/tpg2.20441)
Supplement: Supplementary file 1 [file TPG2-17-e20441-s001.docx]

**A chromosome-level genome of mango exclusively from long-read sequence data**

Upendra Kumari Wijesundara^1^, Ardashir Kharabian Masouleh^1^, Agnelo Furtado^1^, Natalie L Dillon^2^, Robert J Henry^1,3^

^1^ Queensland Alliance for Agriculture and Food Innovation, University of Queensland, Brisbane, 4072 Australia

^2^ Department of Agriculture and Fisheries, Mareeba, 4880, Australia

^3^ ARC Centre of Excellence for Plant Success in Nature and Agriculture, University of Queensland, Brisbane 4072 Australia

**Supplemental Figures**

**Supplemental Figure S1 K-mer profile (K=17) spectrum generated in the Genome Scope for *M. indica* cv.** '**Irwin**'**.** K-mer profiles reveal the complexity of the genomes. Heterozygous genomes show a distinct bimodal profile and a simple poisson profile represents homozygous genomes. The relative heights of the peaks are proportional to the heterozygosity of the genome. Here, the characteristic bimodal profile indicated that the genome is heterozygous. The heterozygosity was estimated as 1.24% (Figure 1). and the genome size was estimated as 365Mbp.

**Supplemental Figure S2 Alignment of (a) *M. indica* cv.** '**Alphonso**' **genome vs** '**Irwin**' **collapsed assembly, (b)** '**Irwin**' **collapsed genome (20 chromosomes) vs** '**Irwin**' **hap1 assembly, (c)** '**Irwin**' **collapsed genome (20 chromosomes) vs** '**Irwin**' **hap2 assembly.**

**Supplemental Figure S3 Alignments between contigs in chromosomes 8, 11, and 19 conducted in CLC-Genomic Workbench**. A: alignment between the contigs ptg000014l and ptg000079l in chromosome 8; B: alignment between the contigs ptg000015l and ptg000035l in chromosome 11; C: alignment between the contigs ptg000813l and ptg000020l in chromosome 19. Here, regions aligned between two contigs have the same repetitive sequence. Among the two contigs in each alignment, the contig on the top is considered as the reference and the contig at the bottom is considered as the query. Colours indicate the sequence similarities/differences between two contigs. Green: both reference and query sequence have the identical nucleotide sequence; Red: Nucleotides are different between two contigs and bottom/query sequence has nucleotide Adenine (A); Blue: Nucleotides are different between two contigs and bottom/query sequence has nucleotide Cytosine (C); Yellow: Nucleotides are different between two contigs and bottom/query sequence has nucleotide Guanine (G); Yellowish green: Nucleotides are different between two contigs and bottom/query sequence has nucleotide Thymine (T).

**Supplemental Figure S4 Alignment of smaller contigs ranged between 16-1000Kb to chloroplast genome (a), mitochondrial genome (b), and ribosomal RNA repeats (c).**

**Supplemental Figure S5 Summary of functional annotation for (a) collapsed, (b) Hap1, (c) Hap2 genomes and top hit species distribution for BLAST hits in (d) collapsed, (e) Hap1, (f) Hap2 genomes.** A blastx search with an e-value cut-off of 1e-3 generated 40,679 CDS sequences (33,013 genes) having BLAST hits whereas hap1 and hap2 had 40,115 (32,576 genes) and 39,333 (31,690 genes) CDS sequences, respectively. For all three genomes, the largest number of BLAST hits were from *M. indica* and 32, 689 (76.12%), 32,140 (76.03%), and 31,667(77.33%) genes were annotated in the collapsed, hap1 and hap2 genomes respectively with at least one gene ontology (GO) term.

**Supplemental Figure S6 The coding potential assessment of CDS sequences that did not give a blast hit during functional annotation in the collapsed genome, hap1, and hap2** using (a) *Arabidopsis thaliana,* (b) *Manifera indica* and as model species. The analysis identified that 1,946 (80.4%), 2,005 (87.6%), and 1512 (88.0%) CDS sequences in respective collapsed, hap1 and hap2 genomes, which did not show any BLAST hits had coding potential when *M. indica* was used as the model. With *Arabidopsis thaliana,* the coding potential of the CDS was higher for all three genomes indicating 99.3%, 98.78%, and 99.07% of the CDS sequences showing coding potential in collapsed, hap1 and hap2 genomes.

**Supplemental Figure S7 Venn diagram displaying the shared and unique gene clusters among** '**Irwin**' **collapsed genome, Hap1, and Hap2.** Comparison among the three genomes ('Irwin' collapsed, hap1 and hap2) produced a total of 28,277 clusters from which 4,596 clusters were shared at least among two assemblies. A total of 88, 83, and 78 clusters were unique to the 'Irwin' collapsed genome, hap1 and hap2, respectively, including 687, 510, and 460 genes in the respective clusters. Furthermore, 1048, 936, and 817 singletons (genes which do not belong to any cluster but are unique in the genome) were identified in collapsed, hap1 and hap2 genomes, respectively, including a total of 1735, 1446, and 1223 unique genes in the respective genomes. GO analysis revealed that cellular processes is the biological process to which most of the unique genes of the three genomes were associated with while majority of the genes were related to binding function and catalytic activity in terms of molecular process. KEGG pathway analysis revealed that most of the unique genes/sequences in the collapsed genome were associated with purine metabolism followed by plant-pathogen interaction and thymine metabolism. In hap1, the highest number of unique genes were related to purine metabolism, where thymine and alpha-Linolenic acid metabolism were the two pathways involving the second and third highest number of genes. Furthermore, in hap2, the highest number of genes was involved in purine metabolism followed by thymine metabolism and the Ras signalling pathway.

**Supplemental Figure S8 Functions of unique genes obtained from gene family analysis with related to biological process and molecular function.** In the collapsed genome, cellular processes and binding functions were the biological process and molecular function to which the highest number of genes were related. In both hap1 and hap2, most of the unique genes were related to cellular processes and catalytic activity in terms of biological process and molecular function, respectively. Many of the other unique genes in the collapsed genome were related to metabolic processes, response to stimuli, biosynthesis process and catalytic activity. In hap1, organic substance and cellular metabolic process, response to stimuli and organic cyclic compound, ion and cation binding were the other abundant GO terms while for hap 2, they were organic substance, organonitrogen compound, and cellular metabolic process, hydrolase activity, protein and ion binding.

**Supplemental Tables**

**Supplemental Table S1 Summary of sequence data generated by two PaBio SMRT cells.**

| **Parameter** | **SMRT cell 1** | **SMRT cell 2** |
| --- | --- | --- |
| HiFi reads | 2,267,089 | 2,202,557 |
| HiFi Yield (bp) | 37,701,344,156 | 37,045,457,789 |
| HiFi Read Length (mean, bp) | 16,629 | 16,819 |
| HiFi Read Quality (median) | Q34 | Q34 |
| Coverage (per SMRT cell) | 103.28 | 101.49 |

**Supplemental Table S2 Assembly and annotation statistics of *M. indica* cv.** '**Irwin**'

| Statistic | Collapsed | Haplotype 1 | Haplotype 2 |
| --- | --- | --- | --- |
| Assembly |  |  |  |
| Total number of contigs | 4,642 | 4,711 | 1,515 |
| Total length (bp) | 556,539,885 | 543,231,508 | 432,295,890 |
| Longest contig (bp) | 28,791,319 | 28,791,319 | 25,591,877 |
| GC % | 36.50 | 36.52 | 34.77 |
| Contig N50 (bp) | 14,984,773 | 13,214,860 | 15,449,518 |
| Contig L50 | 14 | 16 | 12 |
| Complete BUSCO (%) (Viridiplantae, N=425) | 100 | 98.1 | 99.0 |
| Complete & single copy BUSCOS (%) | 89.9 | 88.0 | 89.4 |
| Complete & duplicated BUSCOS (%) | 10.1 | 10.1 | 9.6 |
| Fragmented BUSCOS (%) | 0.0 | 0.7 | 0.0 |
| Missing BUSCOS (%) | 0.0 | 1.2 | 1.0 |
| Annotation |  |  |  |
| Alignment rate of RNA-seq reads to the masked genome (%) | 76.41 | 75.51 | 77.01 |
| Annotation completeness (N=425, %) | 99.6 | 97.6 | 98.6 |
| Number of protein-coding genes | 35,220 | 34,659 | 33,230 |
| Number of CDS | 42,973 | 42,268 | 40,947 |
| Number of proteins | 42,973 | 42,268 | 40,947 |
| Number of exons | 260,047 | 254,996 | 255,344 |
| Number of single exon gene | 6,653 | 6,471 | 5,264 |
| Total gene length (bp) | 110,918,018 | 107,399,304 | 108,372,140 |
| Total exon length (bp) | 52,229,406 | 51,040,974 | 50,850,835 |
| mean gene length (bp) | 3,149 | 3,098 | 3261 |
| mean exon length (bp) | 200 | 200 | 199 |
| Longest gene (bp) | 59,081 | 59,081 | 48,708 |
| Shortest gene (bp) | 201 | 201 | 228 |

| chromosome number | Number of contigs | Contig names | Sequence present at the ends to be joined (NCBI Blast results) | Remarks |
| --- | --- | --- | --- | --- |
| 8 | 2 | ptg000014l | *Mangifera indica* 18S ribosomal RNA | Repetitive sequence at the end/ repeated three times This sequence is repeated in ptg000079l throughout the contig |
|  |  | ptg000079l | *Mangifera indica* 18S ribosomal RNA | Repetitive sequence at the beginning This sequence is repeated in ptg000014l at the end |
| 11 | 2 | ptg000015l | *Mangifera indica* 18S ribosomal RNA | Repetitive sequence at the end This sequence is repeated in ptg000035l-RC throughout the contig |
|  |  | ptg000035l | *Mangifera indica* 18S ribosomal RNA | Repetitive sequence at the end This sequence is repeated in ptg000015l at the end |
| 19 | 2 | ptg000020l | *Spondias tuberosa* external transcribed spacer, partial sequence; and 18S ribosomal RNA gene, internal transcribed spacer 1, 5.8S ribosomal RNA gene, internal transcribed spacer 2, and 26S ribosomal RNA gene, complete sequence | Repetitive sequence at the beginning This sequence is repeated in ptg000813l |
|  |  | ptg000813l | *Spondias tuberosa* external transcribed spacer, partial sequence; and 18S ribosomal RNA gene, internal transcribed spacer 1, 5.8S ribosomal RNA gene, internal transcribed spacer 2, and 26S ribosomal RNA gene, complete sequence | Repetitive sequence at the end This sequence is repeated in ptg000020l |

**Supplemental Table S3 Details of the repetitive sequences present in contigs corresponding to Chromosome 8, 11, and 19**

| Chromosome Number | Chromosome ID | Chromosome length | The presence/absence of telomeric repeats | | | Length of the telomere repeat region at the beginning (bp) | Length of the telomere repeat region at the end (bp) | Total length of the telomere sequence (bp) |
| --- | --- | --- | --- | --- | --- | --- | --- | --- |
|  |  |  | **Presence of telomere seq at both ends** | **Presence of telomeres at one end** | **Absence of telomeres at both ends** |  |  |  |
| 1 | NC_058137.1 | 29,456,600 | - | - | √ | 0 | 0 | 0 |
| 2 | NC_058138.1 | 24,397,897 | - | √ | - | 0 | 22,112 | 22,112 |
| 3 | NC_058139.1 | 23,139,393 | - | √ | - | 21,938 | 0 | 21,938 |
| 4 | NC_058140.1 | 21,507,500 | - | - | √ | 0 | 0 | 0 |
| 5 | NC_058141.1 | 21,083,371 | - | - | √ | 0 | 0 | 0 |
| 6 | NC_058142.1 | 18,811,960 | - | - | √ | 0 | 0 | 0 |
| 7 | NC_058143.1 | 20,623,120 | - | - | √ | 0 | 0 | 0 |
| 8 | NC_058144.1 | 18,243,469 |  | √ | - | 10,991 | 0 | 10,991 |
| 9 | NC_058145.1 | 18,233,274 | √ | - | - | 11,846 | 12,545 | 24,391 |
| 10 | NC_058146.1 | 17,652,500 | - | - | √ | 0 | 0 | 0 |
| 11 | NC_058147.1 | 17,144,574 | - | - | √ | 0 | 0 | 0 |
| 12 | NC_058148.1 | 16,029,966 |  | √ | - | 0 | 17,223 | 17,223 |
| 13 | NC_058149.1 | 15,457,994 | √ | - | - | 11,656 | 7,712 | 19368 |
| 14 | NC_058150.1 | 14,810,209 | - | - | √ | 0 | 0 | 0 |
|  |  |  |  |  |  |  |  |  |
| 15 | NC_058151.1 | 14,765,960 | - | - | √ | 0 | 0 | 0 |
| 16 | NC_058152.1 | 13,817,227 | - | √ |  | 0 | 13,281 | 13,281 |
| 17 | NC_058153.1 | 13,506,273 | - | - | √ | 0 | 0 | 0 |
| 18 | NC_058154.1 | 13,371,754 | - | √ | - | 0 | 34,644 | 34,644 |
| 19 | NC_058155.1 | 13,093,066 | - | - | √ | 0 | 0 | 0 |
| 20 | NC_058156.1 | 12,294,262 | - | - | √ | 0 | 0 | 0 |

**Supplemental Table S4** **Summary of telomeric repeats present in the reference genome currently available for mango (*M. indica* cv.** '**Alphonso**'**)**

**Supplemental Table S5: Details of contigs in the collapsed genome and two haplotypes**

| **Chromosome number** | **Collapsed assembly** | | | | **Haplotype 1** | | | | **Haplotype 2** | | | |
| --- | --- | --- | --- | --- | --- | --- | --- | --- | --- | --- | --- | --- |
|  | **Number of contigs** | **Contig name** | **Contig length (bp)** | **Total length (bp)** | **Number of contigs** | **Contig name** | **Contig length (bp)** | **Total length (bp)** | **Number of contigs** | **Contig name** | **Contig length (bp)** | **Total length (bp)** |
| 1 | 1 | ptg000007l | 28,791,319 | 28,791,319 | 1 | h1tg000008l | 28,791,319 | 28,791,319 | 2 | h2tg000005l  h2tg000006l | 23,219,973  4,602,421 | 27,822,494 |
| 2 | 1 | ptg000023l | 25,591,877 | 25,591,877 | 8 | h1tg000097l  h1tg000095l  h1tg000030l  h1tg000025l  h1tg000033l  h1tg000032l  h1tg000062l  h1tg000122l | 799,828  663,840  6,318,391  9,622,730  976,291  2,831,100  378,101  415,428 | 22,006,409 | 1 | h2tg000016l | 25,591,877 | 25,591,877 |
| 3 | 1 | ptg000006l | 22,617,554 | 22,617,554 |  | h1tg000007l | 22,617,554 | 22,617,554 | 4 | h2tg000003l  h2tg000045l  h2tg000035l  h2tg000044l | 15,999,568  4,138,592  915,246  886,388 | 21,940,194 |
| 4 | 1 | ptg000011l | 21,893,517 | 21,893,517 | 1 | h1tg000012l | 21,770,901 | 21,770,901 | 1 | h2tg000012l | 21,111,872 | 21,111,872 |
| 5 | 1 | ptg000010l | 20,095,946 | 20,095,946 | 1 | h1tg000011l | 20,095,946 | 20,095,946 | 1 | h2tg000002l | 20,115,022 | 20,115,022  18,367,742 |
| 6 | 1 | ptg000019l | 18,861,676 | 18,861,676 | 1 | h1tg000021l | 18,861,676 | 18,861,676 | 1 | h2tg000024l | 18,367,742 |  |
| 7 | 1 | ptg000012l | 22,062,971 | 22,062,971 | 2 | h1tg000028l  h1tg000013l | 6,753,988  14,726,911 | 21,480,999 | 3 | h2tg000033l  h2tg000008l  h2tg000036l | 5,741,954  15,962,511  486,244 | 22,190,909 |
| 8 | 2 | ptg000014l  ptg000079l | 18,025,865  441,791 | 18,467,756 | 2 | h1tg000015l  h1tg000086l | 18,025,865  441,791 | 18,467,756 | 2 | h2tg000004l  h2tg000054l | 17,536,172  629,290 | 18,165,562 |
| 9 | 1 | ptg000009l | 18,285,638 | 18,285,638 | 1 | h1tg000010l | 18,285,638 | 18,285,638 | 3 | h2tg000013l  h2tg000020l  h2tg000046l | 7,437,796  6,360,514  3,458,647 | 17,257,157 |
| 10 | 1 | ptg000031l | 19,503,959 | 19,503,959 | 1 | h1tg000031l | 17,061,531 | 17,061,531 | 1 | h2tg000027l | 19,503,959 | 19,503,959 |
| 11 | 2 | ptg000015l  ptg000035l | 17,012,741  2,464,684 | 19,477,525 | 2 | h1tg000016l  h1tg000040l | 17,012,741  2,458,322 | 19,471,163 | 1 | h2tg000022l | 16,582,699 | 16,582,699 |
| 12 | 1 | ptg000004l | 16,091,751 | 16,091,751 | 1 | h1tg000005l | 16,091,751 | 16,091,751 | 1 | h2tg000007l | 15,803,983 | 15,803,983 |
| 13 | 1 | ptg000018l | 14,984,773 | 14,984,773 | 1 | h1tg000019l | 14,984,773 | 14,984,773 | 1 | h2tg000023l | 14,854,956 | 14,854,956 |
| 14 | 1 | ptg000002l | 14,459,570 | 14,459,570 | 1 | h1tg000003l | 14,459,570 | 14,459,570 | 2 | h2tg000055l  h2tg000001l | 1,610,658  12,444,720 | 14,055,478 |
| 15 | 1 | ptg000028l | 15,449,518 | 15,449,518 | 7 | h1tg000002l  h1tg000358l  h1tg000156l  h1tg000098l  h1tg000149l  h1tg000029l  h1tg000050l | 7,004,884  275,559  208,612  1,493,958  393,993  1,538,843  1,454,206 | 12,370,655 | 1 | h2tg000019l | 15,449,518 | 15,449,518 |
| 16 | 1 | ptg000013l | 14,365,229 | 14,365,229 | 1 | h1tg000014l | 14,365,229 | 14,365,229 | 1 | h2tg000021l | 13,355,921 | 13,355,921 |
| 17 | 1 | ptg000039l | 13,854,346 | 13,854,346 | 1 | h1tg000045l | 13,854,346 | 13,854,346 | 2 | h2tg000037l  h2tg000053l | 10,069,979  2,086,453 | 12,156,532 |
| 18 | 1 | ptg000003l | 13,214,860 | 13,214,860 | 1 | h1tg000004l | 13,214,860 | 13,214,860 | 1 | h2tg000010l | 12,798,669 | 12,798,669 |
| 19 | 2 | ptg000020l  ptg000813l | 13,498,536  113,153 | 13,611,789 | 4 | h1tg000802l  h1tg000035l  h1tg000041l  h1tg000022l | 113,153  5,461,778  4,311,653  3,164,490 | 13,051,374 | 3 | h2tg006013l  h2tg000061l  h2tg000014l | 35,844  1290492  13985328 | 15,311,864 |
| 20 | 1 | ptg000008l | 12,942,385 | 12,942,385 | 1 | h1tg000009l | 12,942,385 | 12,942,385 | 2 | h2tg000009l  h2tg000028l | 4,813,640  7,395,613 | 12,209,353 |

**Supplemental Table S6: Details of chromosomes selected from haplotypes to obtain collapsed assembly.**

| **Chr Number** | **Chromosome Size (Mb)** | | | **Selected chromosome/visually seen chromosome selected for a collapsed genome from haplotype1/haplotype2** | **Chromosome selected from haplotype1/haplotype2 had higher length** |
| --- | --- | --- | --- | --- | --- |
|  | **Collapsed** | **Haplotype 1** | **Haplotype 2** |  |  |
| 1 | 28,791,319 | 28,791,319 | 27,822,494 | Hap1 | √ |
| 2 | 25,591,877 | 22,011,309 | 25,591,877 | Hap2 | √ |
| 3 | 22,617,554 | 22,617,554 | 21,915,575 | Hap1 | √ |
| 4 | 21,893,517 | 21,770,901 | 21,111,872 | Similar to Hap1 | collapsed: size higher than Hap1 and Hap2 |
| 5 | 20,095,946 | 20,095,946 | 20,115,022 | Hap1 | √ |
| 6 | 18,861,676 | 18,861,676 | 18,367,742 | Hap1 | √ |
| 7 | 22,062,971 | 21,480,999 | 22,190,909 | - | collapsed: size ranges in between the sizes of Hap1 and Hap2 |
| 8 | 18,467,756 | 18,467,756 | 18,165,562 | Hap1 | √ |
| 9 | 18,285,638 | 18,285,638 | 17,257,157 | Hap1 | √ |
| 10 | 19,503,959 | 17,061,531 | 19,503,959 | Hap2 | √ |
| 11 | 19,477,525 | 19,471,163 | 16,582,699 | Similar to Hap1 | collapsed: size higher than Hap1 and Hap2 |
| 12 | 16,091,751 | 16,091,751 | 15,803,983 | Hap1 | √ |
| 13 | 14,984,773 | 14,984,773 | 14,854,956 | Hap1 | √ |
| 14 | 14,459,570 | 14,459,570 | 14,055,478 | Hap1 | √ |
| 15 | 15,449,518 | 12,370,055 | 15,449,518 | Hap2 | √ |
| 16 | 14,365,229 | 14,365,229 | 13,355,921 | Hap1 | √ |
| 17 | 13,854,346 | 13,854,346 | 12,156,532 | Hap1 | √ |
| 18 | 13,214,860 | 13,214,860 | 12,798,669 | Hap1 | √ |
| 19 | 13,611,789 | 13,052,274 | 15,311,864 | Similar to Hap1 | collapsed: size ranges in between the sizes of Hap1 and Hap2 |
| 20 | 12,942,385 | 12,942,385 | 12,209,353 | Hap1 | √ |
| Total | 364,623,959 | 354,243,935 | 354,621,142 |  |  |

**Supplemental Table S7: Repetitive elements in the collapsed genome and two haplotypes**

|  | **Collapsed** | | | **Hap1** | | | **Hap2** | | |
| --- | --- | --- | --- | --- | --- | --- | --- | --- | --- |
| **Type of repeat** | **Number of elements** | **Length of elements (bp)** | **Percentage (%)** | **Number of elements** | **Length of elements (bp)** | **Percentage (%)** | **Number of elements** | **Number of elements** | **Percentage (%)** |
| **Retroelements** | 45028 | 45375598 | 12.44 | 54902 | 52041797 | 14.69 | 52905 | 46514302 | 13.12 |
| **SINEs:** | 2008 | 300431 | 0.08 | 2277 | 265348 | 0.07 | 3987 | 501135 | 0.14 |
| **Penelope:** | 297 | 49398 | 0.01 | 0 | 0 | 0 | 398 | 217228 | 0.06 |
| **LINEs:** | 3289 | 3124718 | 0.86 | 2835 | 2870890 | 0.81 | 2878 | 3226452 | 0.91 |
| CRE/SLACS | 0 | 0 | 0 | 0 | 0 | 0 | 0 | 0 | 0 |
| L2/CR1/Rex | 0 | 0 | 0 | 0 | 0 | 0 | 0 | 0 | 0 |
| R1/LOA/Jockey | 0 | 0 | 0 | 0 | 0 | 0 | 0 | 0 | 0 |
| R2/R4/NeSL | 0 | 0 | 0 | 0 | 0 | 0 | 0 | 0 | 0 |
| RTE/Bov-B | 0 | 0 | 0 | 0 | 0 | 0 | 0 | 0 | 0 |
| L1/CIN4 | 3182 | 3050442 | 0.84 | 2835 | 2870890 | 0.81 | 2878 | 3226452 | 0.91 |
| **LTR elements:** | 39731 | 41950449 | 11.51 | 49790 | 48905559 | 13.81 | 46040 | 42786715 | 12.07 |
| BEL/Pao | 11 | 7416 | 0 | 67 | 12604 | 0 | 60 | 51653 | 0.01 |
| Ty1/Copia | 23570 | 23716416 | 6.5 | 28864 | 25892885 | 7.31 | 28764 | 25634797 | 7.23 |
| Gypsy/DIRS1 | 15270 | 17223910 | 4.72 | 19800 | 22049901 | 6.22 | 15692 | 15871837 | 4.48 |
| Retroviral | 79 | 38902 | 0.01 | 0 | 0 | 0 | 824 | 326590 | 0.09 |
| **DNA transposons** | 34023 | 15462750 | 4.24 | 35323 | 17426636 | 4.92 | 27799 | 14485605 | 4.08 |
| **hobo-Activator** | 7431 | 3925200 | 1.08 | 9205 | 4422815 | 1.25 | 6536 | 3996557 | 1.13 |
| **Tc1-IS630-Pogo** | 164 | 28944 | 0.01 | 0 | 0 | 0 | 431 | 111114 | 0.03 |
| **En-Spm** | 0 | 0 | 0 | 0 | 0 | 0 | 0 | 0 | 0 |
| **MULE-MuDR** | 7791 | 6969763 | 1.91 | 10311 | 9244889 | 2.61 | 8232 | 8014518 | 2.26 |
| **PiggyBac** | 0 | 0 | 0 | 0 | 0 | 0 | 0 | 0 | 0 |
| **Tourist/Harbinger** | 1482 | 1030680 | 0.28 | 1187 | 888387 | 0.25 | er 120 | 77999 | 0.02 |
| **Other (Mirage, P-element, Transib)** | 0 | 0 | 0 | 0 | 0 | 0 | 0 | 0 | 0 |
| **Rolling-circles** | 4325 | 1664318 | 0.46 | 4396 | 2099603 | 0.59 | 3300 | 1060883 | 0.3 |
| **Unclassified:** | 404946 | 108055451 | 29.63 | 372407 | 91958560 | 25.96 | 396770 | 104502419 | 29.47 |
| **Total interspersed repeats** |  | 168943197 | 46.33 | repeats: | 161426993 | 45.57 | repeats: | 165719554 | 46.73 |
| **Small RNA:** | 954 | 2159009 | 0.59 | 532 | 2837149 | 0.8 | 248 | 471636 | 0.13 |
| **Satellites:** | 0 | 0 | 0 | 0 | 0 | 0 | 0 | 0 | 0 |
| **Simple repeats:** | 83779 | 4090640 | 1.12 | 89146 | 4754844 | 1.34 | 84005 | 3951314 | 1.11 |
| **Low complexity:** | 16107 | 784107 | 0.22 | 16041 | 787112 | 0.22 | 15979 | 773645 | 0.22 |
| **bases masked** | - | 177615148 | 48.71 | - | 171905701 | 48.53 | - | 171977032 | 48.5 |

| **Chr Number** | **Collapsed** | | **Hap1** | | **Hap2** | |
| --- | --- | --- | --- | --- | --- | --- |
|  | **Chr size (bp)** | **Number of genes** | **Chr size (bp)** | **Number of genes** | **Chr size (bp)** | **Number of genes** |
| 1 | 28,791,319 | 2,701 | 28,791,319 | 2,720 | 27,822,494 | 2,553 |
| 2 | 25,591,877 | 2,210 | 22,011,309 | 2,084 | 25,591,877 | 2,164 |
| 3 | 22,617,554 | 2,507 | 22,617,554 | 2,496 | 21,915,575 | 2,370 |
| 4 | 21,893,517 | 2,289 | 21,770,901 | 2,236 | 21,111,872 | 2,149 |
| 5 | 20,095,946 | 2370 | 20,095,946 | 2,389 | 20,115,022 | 2,297 |
| 6 | 18,861,676 | 1,778 | 18,861,676 | 1,805 | 18,367,742 | 1,682 |
| 7 | 22,062,971 | 1,998 | 21,480,999 | 1,945 | 22,190,909 | 1,886 |
| 8 | 18,467,756 | 2,141 | 18,467,756 | 2,124 | 18,165,562 | 2,059 |
| 9 | 18,285,638 | 1,880 | 18,285,638 | 1,885 | 17,257,157 | 1,745 |
| 10 | 19,503,959 | 1,807 | 17,061,531 | 1,694 | 19503959 | 1,699 |
| 11 | 19,477,525 | 1,823 | 19,471,163 | 1,867 | 16582699 | 1,460 |
| 12 | 16,091,751 | 1,700 | 16,091,751 | 1,774 | 15803983 | 1,676 |
| 13 | 14,984,773 | 1,394 | 14,984,773 | 1,374 | 14854956 | 1,362 |
| 14 | 14,459,570 | 1,488 | 14,459,570 | 1,457 | 14,055,478 | 1,377 |
| 15 | 15,449,518 | 1,269 | 12,370,055 | 1,056 | 15449518 | 1,226 |
| 16 | 14,365,229 | 1,161 | 14,365,229 | 1,139 | 13355921 | 1,103 |
| 17 | 13,854,346 | 1,278 | 13,854,346 | 1,228 | 12,156,532 | 1,138 |
| 18 | 13,214,860 | 1,177 | 13,214,860 | 1,202 | 12798669 | 1,123 |
| 19 | 13,611,789 | 1,230 | 13,052,274 | 1,160 | 15,311,864 | 1,174 |
| 20 | 12,942,385 | 1,019 | 12,942,385 | 1,024 | 12,209,353 | 987 |
| Total | 364,623,959 | 35,220 | 354,243,935 | 34,659 | 354,621,142 | 33,230 |

**Supplemental Table S8 Number of genes in chromosomes of collapsed genome and haplotypes**

**Supplemental Methods**

**Joining contigs in Irwin haplotype 1 and haplotype 2.**

1. **Identification of contigs corresponding to chromosomes**

Initial contig assemblies for 'Irwin' Haplotype 1 (Hap1) and Haplotype 2 (Hap2) were aligned with the finalized 'Irwin' collapsed genome (20 chromosomes) in D-GENIES to identify contigs corresponding to hap1 and hap2. Contig assemblies were sorted and re-oriented with respect to the Irwin collapsed genome.

2. **Linking contigs to generate complete pseudomolecules for chromosomes.**

- For the chromosomes that required the joining of two contigs only,

1. The presence of repetitive regions and overlapping regions at the ends of the two contigs were examined by NCBI blast, and long-read mapping in the CLC-genomic workbench respectively.
2. When the same repetitive sequence was present (eg: 18S ribosomal RNA repeats), the two contigs were linked by adding 100Ns to represent any missing copies of the repetitive sequence between the two contigs.
3. When the two contigs had an overlapping region, first it was checked whether the overlapping sequence was truly present in the genome by checking the presence of the same sequence in the collapsed genome and in the other haplotype. If the overlapping sequence was not duplicated either in the collapsed genome or in the other haplotype, it was identified as an assembly artifact that had put the same sequence in both contigs. Therefore, the duplicated sequence was removed from one of the contigs and then the two contigs were joined without adding any Ns.
4. When there was a gap between the two contigs and the two ends had sequences coding for different genes (identified by NCBI blast), they were linked by adding 100 Ns to represent the gap present between the two contigs.

- For the chromosomes that required the joining of more than two contigs.

1. After aligning and re-orienting the contigs with the respective chromosome in the collapsed genome, the presence of the same repetitive sequence/ gaps/ overlapping sequences between ends of adjacent contigs was checked.
2. The contigs were joined sequentially using the approach used in joining two contigs as explained above.
3. If the same contig breaks and aligns with two different regions of the same chromosome in the collapsed genome, it was first confirmed whether the contig had two regions belonging to different locations in the chromosome. This was done by aligning the contig with the other haplotype in D-GENIES and by aligning the contig with the chromosome of the collapsed genome in the CLC-genomic workbench. If the contig truly had sequences belonging to different regions in the same chromosome, the exact breakpoint was identified by whole genome alignment/ long-read mapping of the contigs with the respective chromosome in the collapsed genome.

Then after splitting the contig into two, contigs were again joined using the same approach that we used to join two contigs.

**Table 1 Details of the methods used for linking chromosomes in** '**Irwin**' **Hap1 and Hap2**

| **Chr number** | **Hap1** | | | | **Hap2** | | | |
| --- | --- | --- | --- | --- | --- | --- | --- | --- |
|  | **Number of contigs present** | **Contig names in order** | **The presence of overlapping regions/gaps/ repeats at the end of the contigs required joining** | **Method of joining contigs** | **Number of contigs present** | **Contig names in order** | **The presence of overlapping regions/gaps/ repeats at the end of the contigs required joining** | **Method of joining contigs** |
| Chr 01 | 1 | h1tg000008l | - | - | 2 | h2tg000005l  h2tg000006l | A gap | By adding 100Ns |
| Chr 02 | 8 | 1.h1tg000097l  2.h1tg000095l  3.h1tg000030l  4.h1tg000025l  5.h1tg000033l  6.h1tg000032l  7.h1tg000062l  8.h1tg000122l | Gaps were present among all the adjacent contigs. | By adding 100Ns between each of the adjacent two conti1gs | 1 | h2tg000016l | - | - |
| Chr 03 | 1 | h1tg000007l | - | - | 4 | 1.h2tg000003l  2.h2tg000045l-1  3.h2tg45000035l  4.h2tg000044l  5.h2tg000045l-2 | h2tg000045l aligned into two different regions and it was split into two contigs after identifying coordinates (1 and 2) h2tg000045l- had telomeric repeats at the end confirming its location (Figure 1, 2).  There were gaps among the contigs h2tg000003l, h2tg000045l-1, and h2tg000035l and an overlapping region between h2tg000044l and h2tg000045l-2 (Figure 3). | h2tg000003l, h2tg000045l-1, h2tg000035l, and h2tg000044l were joined by adding 100Ns.  h2tg000044l and h2tg000045l-2 were joined without adding Ns after removing the overlapping region from h2tg000044l. |
| Chr 04 | 1 | h1tg000012l | - | - | 1 | h2tg000012l | - | - |
| Chr 05 | 1 | h1tg000011l | - | - | 1 | h2tg000002l | - | - |
| Chr 06 | 1 | h1tg000021l | - | - | 1 | h2tg000024l | - | - |
| Chr 07 | 2 | h1tg000028l  h1tg000013l | A gap | By adding 100Ns | 3 | h2tg000033l  h2tg000008l  h2tg000036l | h2tg000033l and h2tg000008l  had a small gap.  h2tg000008l had 5S rRNA repeats, but h2tg000036l had sequence coding for a signal peptide protein | Three contigs were joined by adding 100Ns in between. |
| Chr 08 | 2 | 1.h1tg000015l  2.h1tg000086l | 18S rRNA repeats at ends of the both contigs | By adding 100Ns | 2 | h2tg000004l  h2tg000054l | 18S rRNA repeats at ends of both contigs | By adding 100Ns |
| Chr 09 | 1 | h1tg000010l | - | - | 3 | h2tg000013l  h2tg000020l  h2tg000046l | Gaps among three contigs | By adding 100Ns |
| Chr 10 | 1 | h1tg000031l | - | - | 1 | h2tg000027l | - | - |
| Chr 11 | 2 | 1.h1tg000016l  2.h1tg000040l | 18S rRNA repeats at ends of the both contigs | By adding 100Ns | 1 | h2tg000022l | - | - |
| Chr 12 | 1 | h1tg000005l | - | - | 1 | h2tg000007l | - | - |
| Chr 13 | 1 | h1tg000019l | - | - | 1 | h2tg000023l | - | - |
| Chr 14 | 1 | h1tg000003l | - | - | 2 | h2tg000055l  h2tg000001l |  |  |
| Chr 15 | 7 | 1.h1tg000002l  2.h1tg000358l  3.h1tg000156l  4.h1tg000098l  5.h1tg000149l  6.h1tg000029l  7.h1tg000050l | Gaps were present among all the adjacent contigs. | By adding 100Ns | 1 | h2tg000019l | - | - |
| Chr 16 | 1 | h1tg000014l | - |  | 1 | h2tg000021l | - | - |
| Chr 17 | 1 | h1tg000045l | - |  | 2 | h2tg000037l  h2tg000053l |  |  |
| Chr 18 | 1 | h1tg000004l | - |  | 1 | h2tg000010l | - | - |
| Chr 19 | 4 | 1.h1tg000802l  2.h1tg000035l  3.h1tg000041l  4.h1tg000022l | Between h1tg000802l and h1tg000035l-rRNA repeats  The ends of h1tg000035l and h1tg000041l joined had different coding sequences.  A gap was present between h1tg000041l and h1tg000022l | By adding 100Ns in between all the contigs | 3 | h2tg006013l  h2tg000061l  h2tg000014l | h2tg006013l has 28S rRNA and h2tg000061l has 18S rRNA repeats.  There is a gap between h2tg000061l and h2tg000014l | By adding 100Ns in between all the contigs |
| Chr 20 | 1 | h1tg000009l | N/A | - | 2 | h2tg000009l  h2tg000028l | A gap | By adding 100Ns |

**Figure 1 Dot plot showing the alignment of contigs of** '**Irwin**' **haplotype 2: chromosome 3.** There were four contigs in haplotype 2 aligned with chromosome 3 of the Irwin collapsed genome. h2tg000045l aligned with two different regions in chromosome 3 of the collapsed genome (ht2g000045l-1, h2tg000045l-2) and h2tg000045l-2 had telomeric repeats at the end of the sequence confirming that it is actually the end region of chromosome 3.

**Figure 2 Whole genome alignment between A:h2tg000003l, B: h2tg000045l, C: h2tg000035l, D: h2tg000044l belongs to chromosome 3 of** '**Irwin**' **haplotype 2 with that of the collapsed genome**. The alignments showed that there are gaps between h2tg000003l and h2tg000045l-1, h2tg000045l-1 and h2tg000035l, h2tg000035l and h2tg000044l. Alignment between h2tg000044l and h2tg000045l-2 showed that there was an overlapping region between these two contigs.

**Figure 3 Alignment of h2tg000044l and h2tg000045-2 via long-read mapping.** Alignment between two contigs enabled finding the exact overlapping region between two contigs. This sequence was not duplicated either in haplotype 1 or in the collapsed genome. Therefore, the overlapping region was removed from h2tg000044l (861,861-886,388bp) and the two contigs were linked without adding Ns.
